# Prebiotic-Empowered Probiotics with Gastrointestinal Stress Resistance for Enhanced Oral Therapy of Immunosuppression

**DOI:** 10.3390/foods15091540

**Published:** 2026-04-29

**Authors:** Xiaomin Chen, Huangxin Zhu, Zuwei Liu, Qianru Zhao, Ying Zhang, Yiqun Wan, Hao Wan

**Affiliations:** 1 State Key Laboratory of Food Science and Resources, Nanchang University, Nanchang 330047, China; chen123xiaomin@163.com (X.C.); zuweiliu@163.com (Z.L.); zqianru0816@163.com (Q.Z.); zhangying908@163.com (Y.Z.); wanyiqun@ncu.edu.cn (Y.W.); 2 Jiangxi Provincial Key Laboratory of Digestive Diseases, Department of Gastroenterology, Jiangxi Clinical Research Center for Gastroenterology, The First Affiliated Hospital, Jiangxi Medical College, Nanchang University, Nanchang 330066, China; 360014250097@email.ncu.edu.cn

**Keywords:** immunosuppression, *Limosilactobacillus reuteri*, β-glucan, biorthogonal engineering

## Abstract

Oral probiotic-based therapy has emerged as a promising solution with multifaceted benefits for immunosuppression treatment. However, their widespread and clinical utility is severely limited by the poor viability of probiotics under harsh gastrointestinal conditions in the intestine. To address these challenges, a probiotic-based biohybrid (Lr@DGN) was bio-orthogonally fabricated by covalently anchoring the prebiotic β-glucan (GN) to the probiotic *Limosilactobacillus reuteri* (Lr). Upon oral administration, Lr@DGN colonized intestines with high survival rates, aided by gastrointestinal stress-shielding of GN, leading to immuno-enhancing effects through combining GN and live Lr. Consequently, in a Cy-induced immunosuppression mouse model, oral administration of Lr@DGN significantly mitigated body weight loss, restored the key immune organ indexes (thymus and spleen), ameliorated Cy-induced damage to the small intestine, enhanced the intestinal immune response, and elevated the serum levels of immunoglobulins IgG and IgA. By integrating the effects of a prebiotic shield and a live probiotic, this biohybrid system offers a promising and translatable approach for managing immunodeficiency and related disorders.

## 1. Introduction

Chemotherapy-induced immunosuppression represents a formidable clinical challenge that extends beyond the intended targeting of malignant cells [1]. Agents such as cyclophosphamide (Cy) cause three characteristic types of damage: systemic loss of leukocytes [2], involution of primary lymphoid organs [3], and compromise of the intestinal epithelial barrier [4]. This damage in gut integrity facilitates microbial translocation and perpetuates a cycle of inflammation and immune dysfunction [5,6], creating a situation that demands simultaneous intervention at both the mucosal and systemic levels. Consequently, an optimal therapeutic strategy must not only promote systemic immune reconstitution but also actively repair the intestinal barrier to restore holistic homeostasis.

Probiotics, particularly lactobacillus strains, emerge as a compelling therapeutic platform inherently capable of addressing this dual mandate. Through colonization, they competitively exclude pathogens and reinforce tight junctions to directly strengthen the gut epithelium [7,8]. Concurrently, their cellular components and metabolites interact with host immune cells to exert broad immunomodulatory effects [9,10]. However, the oral delivery of conventional probiotics is fundamentally undermined by the harsh gastrointestinal environment [11,12]. Gastric acid and bile salts act as a formidable barrier, drastically reducing the viable probiotic count that reaches the intestine, thereby eliminating their therapeutic potential at the intended site of action [13]. Thus, ensuring the survival of probiotics through the gastrointestinal tract is not merely an enhancement but a prerequisite for achieving their dual therapeutic functionality.

Traditional encapsulation methods, such as spray-drying or emulsion, provide a physical barrier at a significant cost [14]. These processes often subject bacterial cells to thermal, osmotic, or shear stresses that can irreversibly damage their viability and biological activity—an undesirable side effect of the protective measures [15,16]. This limitation underscores the need for a protective approach that is inherently gentle on living cells. We therefore posit that an ideal strategy should integrate therapeutic functionality into the protective matrix, rather than using it solely as a passive barrier. This “functional protection” strategy thus requires a material that not only forms a stable, biocompatible shield under mild conditions but also resists gastrointestinal degradation and, critically, actively participates in therapeutic outcomes by promoting intestinal repair and immune activation upon arrival.

Guided by these criteria, β-glucan (GN) is logically identified as the material of choice. As a natural polysaccharide, its robust structure provides inherent resistance to gastric acid and bile salt digestion [17], fulfilling the prerequisite for physical protection. More importantly, upon arrival in the intestine, GN exerts a dual therapeutic action. On one hand, it serves as a prebiotic, selectively fermented by commensal bacteria into short-chain fatty acids (SCFAs) [18,19]—key metabolites renowned for promoting epithelial cell proliferation [20], enhancing mucus secretion [21], and strengthening the gut barrier [22]. On the other hand, GN is a potent biological response modifier that can directly bind to immune receptors such as dectin-1 on macrophages and dendritic cells, triggering signaling cascades that promote anti-inflammatory cytokine profiles and stimulate innate immune defenses [23]. This unique combination of properties positions GN not merely as a shield, but as a therapeutic payload that synergizes with the probiotic core. As such, its breakdown products not only create a favorable niche for probiotic colonization but also exert immunostimulatory effects that run in parallel with the probiotic’s own immunomodulation.

The final challenge lies in the precise, gentle, and stable assembly of this functional GN layer onto individual probiotic cells without compromising their vitality. Conventional conjugation chemistry is often incompatible with living systems due to harsh conditions or non-specific reactions [24]. Here, bio-orthogonal click chemistry provides an elegant solution. This technique allows for specific, covalent linkage under physiological conditions, acting as a molecular “click” to seamlessly install the GN armor onto the bacterial surface without inflicting damage [25,26]. It is the enabling technology that makes this rational design physically achievable.

Although the potential of combining probiotics with β-glucan in intestinal and immune restoration has been widely recognized, two critical issues still require further resolution. One problem lies in the fact that many existing delivery systems either reduce probiotic viability during formulation or cannot maintain adequate survival throughout the gastrointestinal tract, which in turn weakens their therapeutic efficacy. Another limitation is that no available strategy has yet incorporated a dual-functional protective layer capable of preserving probiotic viability while simultaneously participating in intestinal barrier repair and immune modulation through a chemically defined and gentle approach. Under these conditions, a method integrating gentle surface engineering with therapeutically active protection has still not been developed. To address these gaps, we report the design of Lr@DGN, a biohybrid therapeutic synthesized by conjugating dibenzocyclooctyne (DBCO)-modified GN nanoparticles (DGN) onto azide-functionalized *Limosilactobacillus reuteri* (Lr) via copper-free click chemistry. We hypothesize that Lr@DGN will function as a shielded, micro-factory, wherein the DGN shell protects Lr during gastrointestinal transit and subsequently contributes its own barrier repair and immunostimulatory effects upon reaching the intestine, thereby augmenting the probiotic actions of the viable Lr core. This work details the synthesis and characterization of Lr@DGN, demonstrates its enhanced survival under gastrointestinal stresses, establishes its biocompatibility in vivo, and demonstrates its superior efficacy in alleviating Cy-induced immunosuppression in mice, outperforming all control treatments. Our study establishes a versatile platform for engineering advanced living therapeutics through rational, function-driven design, filling the identified gap in gentle yet functionally active probiotic surface engineering.

## 2. Materials and Methods

### 2.1. Materials

Xinyan Bomei Biotechnology Co., Ltd. (Xi’an, China) provided the 3-Azido-D-alanine HCl. Megazyme Co., Ltd. (Bray, Ireland) offered the β-Glucan. Cyclophosphamide (Cy) and dibenzocyclooctyne-amine (DBCO-NH_2_) were procured from Aladdin Biochemical Technology Co., Ltd. (Shanghai, China). Click Chemistry Tools (Scottsdale, AZ, USA) was the source of AZDye™ 647 DBCO. Sigma-Aldrich (St. Louis, MO, USA) supplied the following reagents: N-(3-Dimethylaminopropyl)-N’-ethylcarbodiimide hydrochloride (EDC), N-hydroxysuccinimide (NHS), and lipopolysaccharide (LPS). Shanghai Keshun Co., Ltd. (Shanghai, China) provided the enzyme-linked immunosorbent assay (ELISA) kits for immunoglobulin G (IgG) and immunoglobulin A (IgA). MRS broth (catalog No. HB0384-1) was obtained from Hopebio Co., Ltd. (Qingdao, China). Analytical grade reagents were used for all other experiments, without further modification.

### 2.2. Preparation of DGN

Following our previously established protocol [27], DGN was synthesized. Briefly, carboxylated GN (30 mg) was first dissolved in MES buffer (pH 6.5) and stirred at 25 °C for 10 min. Subsequently, EDC (4 mg) was introduced, and the reaction was allowed to proceed for 30 min. After that, NHS (4 mg) along with DBCO-NH_2_ (5 mg) were blended into the mixture, which was then stirred overnight. Following neutralization, the mixture was purified by dialysis against distilled water. The resultant product was then lyophilized to yield DGN. Using a UV-Vis absorbance-based assay, the conjugation efficiency of DBCO was determined, with the substitution degree calculated as 0.172 µmol DBCO per mg DGN. The overall yield of DGN, expressed as the mass ratio of the lyophilized product to the initial mass of carboxylated GN, was 83.3%.

### 2.3. Bacterial Strains and Growth Parameters

The Guangdong Microbial Culture Collection Center (GDMCC, Guangzhou, China) provided the Lr strain (GDMCC No. 1.1805). The Lr strain was initially streaked onto MRS agar plates and incubated at 37 °C for 24 h to obtain isolated colonies. Afterward, a single colony was transferred into liquid MRS broth and cultured overnight (12–16 h) at 37 °C with shaking at 180 rpm to prepare the seed culture. For experimental use, the seed culture was diluted to a 4% (*v*/*v*) inoculum in fresh MRS broth and incubated under the same conditions until the culture reached the desired growth phase.

### 2.4. Fabrication and Characterization of Lr@DGN

Lr was cultured in MRS broth supplemented with 6 mM 3-Azido-D-alanine HCl at 37 °C and 200 rpm for 12 h to obtain Lr-N_3_ through metabolic incorporation of azide groups onto the bacterial surface. After centrifugation (approximately 3500× *g*, 3 min) using a centrifuge (Thermo Fisher Scientific, Waltham, MA, USA), the pellet was washed twice with PBS to remove excess azido-alanine. Equal volumes of bacterial suspension were used in all experimental conditions. For click conjugation, DGN was added at a final concentration of 0.8 mg/mL, and the mixture was incubated at 37 °C and 200 rpm for 12 h. Lr@DGN was then collected by centrifugation and washed three times with PBS to remove unbound DGN. For the physical mixture control group (Lr + DGN), DGN was combined with unmodified Lr at the same concentrations applied in Lr@DGN preparation, after which the mixture was incubated under identical conditions (37 °C, 200 rpm, 12 h) and subsequently centrifuged and washed with PBS to eliminate unbound DGN. The conjugation yield was calculated as the CFU count after conjugation divided by the initial CFU count.

Bacterial samples of Lr and Lr@DGN were used for morphological characterization. The samples were fixed in 2.5% glutaraldehyde in PBS overnight at 4 °C, followed by three washes with PBS and stepwise dehydration in ethanol solutions of 50%, 70%, 80%, 90%, and 100%, each for 15 min. After dehydration, the samples were dried under vacuum and sputter-coated with a 10 nm gold layer. Surface morphology was observed using a Hitachi Regulus 8100 scanning electron microscope (Hitachi High-Technologies, Minato, Japan), and the images obtained were used for analysis.

For validation of azide incorporation, Lr-N_3_ was labeled with DBCO-AF647 (1 μg/mL) in PBS at 37 °C for 4 h and washed twice with PBS before fluorescence analysis by flow cytometry (CytoFLEX, Indianapolis, IN, USA). Unmodified Lr was used as the negative control.

Stability assessment of Lr@DGN: To evaluate the colloidal stability of the DGN coating, Lr@DGN was resuspended in PBS (pH 7.4) at a concentration of approximately 1 × 10^8^ CFU/mL and incubated at 37 °C with gentle shaking (100 rpm). At 0 and 24 h, aliquots were collected and the hydrodynamic diameter was measured using a Zetasizer (Malvern, UK).

### 2.5. In Vitro Assessment of Gastrointestinal Tolerance of Lr@DGN

The in vitro gastrointestinal resistance of Lr and Lr@DGN was evaluated using three different assays, all performed at 37 °C with shaking at 180 rpm. In the gastric fluid tolerance assay, samples were incubated in simulated gastric fluid (SGF) for 2 h. SGF was prepared by dissolving 0.2% (*w*/*v*) NaCl and 0.32% (*w*/*v*) pepsin, and then adjusting the pH to 2.5 with HCl, as described in previous studies [28]. For the bile salt tolerance assay, samples were incubated for 2 h in phosphate-buffered saline (PBS) containing 4% (*w*/*v*) bile salts, a concentration chosen based on earlier research on probiotic tolerance to gastrointestinal stress [27]. The sequential gastrointestinal tolerance assay began with 2 h of incubation in SGF (pH 2.5), followed by centrifugation (approximately 3500× *g* for 3 min), washing with PBS, and resuspension in simulated intestinal fluid (SIF) for an additional 2 h. The SIF was prepared with 0.68% (*w*/*v*) KH_2_PO_4_, adjusted to pH 6.8 using NaOH, and supplemented with 1% (*w*/*v*) pancreatin and 4% (*w*/*v*) bile salts, following the protocol [28]. Samples were collected at time points of 0, 1, and 2 h for the gastric fluid, at 0 and 2 h for bile salt assays, and at 0 and 4 h for the sequential assay. At each time point, 100 μL aliquots were taken for viability analysis. After centrifugation, the pellets were washed with PBS, serially diluted, and plated on MRS agar for colony counting. All experiments were conducted in triplicate.

### 2.6. Experimental Animals

GemPharmatech Co., Ltd. (Nanjing, China) provided the specific pathogen-free (SPF) female BALB/c mice (6–8 weeks old, 18 ± 2 g) used in this study; all procedures involving animals having been approved by the Animal Ethics Committee of Nanchang University (approval no. NCULAE-202601010099) and conducted in accordance with relevant guidelines. Mice were acclimatized under controlled conditions (temperature: 24 ± 2 °C, humidity: 55 ± 10%, 12 h light/dark cycle) with ad libitum access to food and water for one week prior to experiments.

### 2.7. In Vivo Toxicity Analysis

Mice were randomized into two experimental groups (n = 3 per group). One group underwent daily oral gavage of PBS (control), while the other received Lr@DGN (1 × 10^10^ CFU per dose in PBS) for seven consecutive days. At the endpoint, blood and major organs were collected for serum biochemistry and pathological analysis.

### 2.8. Therapeutic Effect of Lr@DGN on Cy-Induced Immunosuppression Model

Following acclimatization, mice were randomly assigned to six experimental groups (n = 5 per group): (1) Normal (saline injection + PBS gavage), (2) Model (Cy injection + PBS gavage), (3) DGN (Cy injection + free DGN gavage), (4) Lr (Cy injection + free Lr gavage, 1 × 10^10^ CFU/dose), (5) Lr + DGN (Cy injection + physical mixture of Lr and DGN), and (6) Lr@DGN (Cy injection + Lr@DGN gavage, 1 × 10^10^ CFU-equivalent/dose). For the groups treated with bacteria, including Lr, Lr + DGN, and Lr@DGN, fresh suspensions were prepared on each day of dosing. A standardized preparation procedure had been validated before the animal experiments by CFU plate counting, allowing the dosing concentration to remain consistent throughout the study. Briefly, free Lr, Lr@DGN, or the physical mixture was cultured overnight for 12–16 h at 37 °C with shaking at 180 rpm, followed by centrifugation at approximately 3500× *g* for 3 min, two washes with sterile PBS, and resuspension in PBS to a fixed volume. Preliminary testing confirmed that this procedure could reproducibly yield 1 × 10^10^ CFU per 200 μL dose. This was determined by serial dilution and plating on MRS agar in three independent replicates. During the in vivo experiments, all batches were prepared with the same protocol and used within 2 h. Additional spot-check CFU plate counting was carried out on selected days, usually every third day.

Cy (80 mg/kg body weight) was administered intraperitoneally for three consecutive days to induce immunosuppression. Between days 3 and 9, each group received their respective daily oral treatments. Mice fasted overnight after the final treatment and euthanized. The small intestine, spleen, and thymus were collected, weighed, and either snap-frozen or fixed for subsequent analysis.

### 2.9. Body Weight Monitoring and Immune Organ Index Analysis

Body weight was recorded daily throughout the study. After euthanasia, the thymus and spleen were gently removed, rinsed with saline, blotted dry, and weighed. The organ index was determined using the following formula:

$$Organ\;Index=\frac{Organ\;Weight\;(g)}{Body\;Weight\;(g)}\;\times 100\%$$

### 2.10. Histopathological Analysis of Intestinal Tissue

Small intestinal segments were fixed in 4% paraformaldehyde (pH 7.4) at 4 °C for 24 h, then dehydrated through graded ethanol solutions of 70%, 80%, 95%, and 100%, cleared in xylene, and embedded in paraffin wax. Sections were cut to 4 μm thickness. During hematoxylin and eosin (H&E) staining, the paraffin sections were deparaffinized in xylene twice, rehydrated through descending concentrations of ethanol, and rinsed with distilled water. The sections were stained with hematoxylin for 5 min, differentiated in 0.3% acid alcohol containing 1% HCl in 70% ethanol for 3 s, returned to tap water for 10 min to complete bluing, and then counterstained with eosin for 2 min, following standard histological procedures [29]. After another round of dehydration in graded ethanol and clearing in xylene, the sections were sealed with neutral resin. Morphological observation was carried out under a light microscope at 200× magnification.

### 2.11. Alcian Blue/Periodic Acid–Schiff (AB-PAS) Protocol

Intestinal paraffin sections were subjected to Alcian blue/periodic acid–Schiff (AB-PAS) staining to visualize goblet cells and examine mucin production. Staining was carried out using an AB-PAS staining kit following the manufacturer’s instructions, with slight adjustments to the procedure. After deparaffinization and rehydration as described in Section 2.10, the sections were immersed in Alcian blue solution at pH 2.5 for 30 min at room temperature and then rinsed in running tap water for 5 min. The sections were subsequently treated with 0.5% periodic acid solution for 10 min, rinsed with distilled water, and incubated in Schiff’s reagent for 15 min in the dark, following standard histochemical procedures [30]. After an additional 10 min wash in running tap water, hematoxylin was applied for 1 min as a counterstain. The sections were then dehydrated, cleared, and mounted with neutral resin. Alcian blue-positive goblet cells, indicating acidic mucins, appeared blue, whereas PAS-positive goblet cells, representing neutral mucins, appeared magenta under a light microscope.

### 2.12. Immunofluorescence Staining for T Cells

CD4^+^ and CD8^+^ T cell populations in the small intestine were examined by immunofluorescence staining. After deparaffinization and rehydration as described in Section 2.10, antigen retrieval was carried out in citrate buffer (10 mM, pH 6.0) by heating the sections at 95 °C for 20 min and then allowing them to cool to room temperature. The sections were washed three times with PBS and blocked for 1 h at room temperature in 5% bovine serum albumin prepared in PBS containing 0.1% Tween-20 to reduce non-specific binding. Rabbit anti-CD4 (1:200) and rat anti-CD8 (1:200), diluted in blocking buffer, were applied overnight at 4 °C. After three further washes with PBS, the sections were incubated for 1 h at room temperature in the dark with Alexa Fluor 488-conjugated goat anti-rabbit IgG (1:500) and Alexa Fluor 594-conjugated goat anti-rat IgG (1:500), following standard immunofluorescence procedures for paraffin sections [30]. The nuclei were counterstained with 4′,6-diamidino-2-phenylindole (DAPI, 1 μg/mL) for 10 min. After a final wash, the sections were mounted with anti-fade mounting medium, and fluorescence images were collected under a fluorescence microscope using the appropriate filter sets.

### 2.13. Serum Biomarker Quantification by ELISA

Whole blood was collected after euthanasia and allowed to clot at room temperature for 2 h. Serum was isolated by centrifugation at 3000× *g* for 15 min at 4 °C, then stored at −80 °C until analysis. The concentrations of immunoglobulin A (IgA) and immunoglobulin G (IgG) in serum were measured using commercial enzyme-linked immunosorbent assay (ELISA) kits in accordance with the manufacturer’s instructions.

### 2.14. Statistical Analysis

Data are presented as mean ± SD. Student’s *t*-test was applied for comparisons between two groups, and one-way ANOVA for comparisons among three or more groups. For one-way ANOVA that yielded a significant result, Dunnett’s multiple comparisons test were employed for post hoc analysis, comparing all experimental groups against the control group. * *p* < 0.05, ** *p* < 0.01, *** *p* < 0.001, and **** *p* < 0.0001 were considered statistically significant.

## 3. Results

### 3.1. Bio-Orthogonal Preparation of Lr@DGN and Its Characterization

To assess whether DBCO conjugation was successful, the substitution degree was determined first. Under UV-Vis spectrophotometry, and with unmodified GN used as the background control, the DBCO substitution degree was calculated to be 0.172 μmol/mg DGN on the basis of the molar extinction coefficient of DBCO (12,000 M^−1^ cm^−1^). The overall yield of DGN after dialysis and lyophilization reached 83.3%, this value being derived from the mass ratio of the lyophilized product to the initial carboxylated GN. The surface charge of DGN was further characterized through Zeta potential analysis, and Figure S1 shows that DGN exhibited a Zeta potential of −36.98 mV, lower than carboxylated GN (−23.68 mV).

Lr-N_3_ was labeled with a DBCO-AF647 fluorescent probe to confirm successful azide incorporation onto the bacterial surface. Compared with unmodified Lr, Lr-N_3_ showed a distinct fluorescence shift in flow cytometry analysis, and the mean fluorescence intensity was approximately 2.85-fold higher (Figure S2).

To demonstrate our concept, we selected Lr as a model probiotic. Various strains of Lr are known to regulate immune responses [31] and improve intestinal barrier integrity [32], two properties closely related to the relief of immunosuppression and the recovery of the mucosa. The strain applied in the present work was GDMCC No. 1.1805, which was obtained from the Guangdong Microbial Culture Collection Center and selected as a representative strain of this species. To prepare Lr@DGN, the probiotic surface was first decorated with azide groups (Lr-N_3_) through metabolic incorporation of 3-Azido-D-alanine HCl into the peptidoglycan layer, as described in Section 2.4. Subsequently, covalent coating of DGN onto Lr was achieved via copper-free click chemistry between azide and DBCO groups (see DBCO modification of GN in “Section 2”), yielding a bio-orthogonal and efficient protective layer (Lr@DGN).

**Figure 1:**
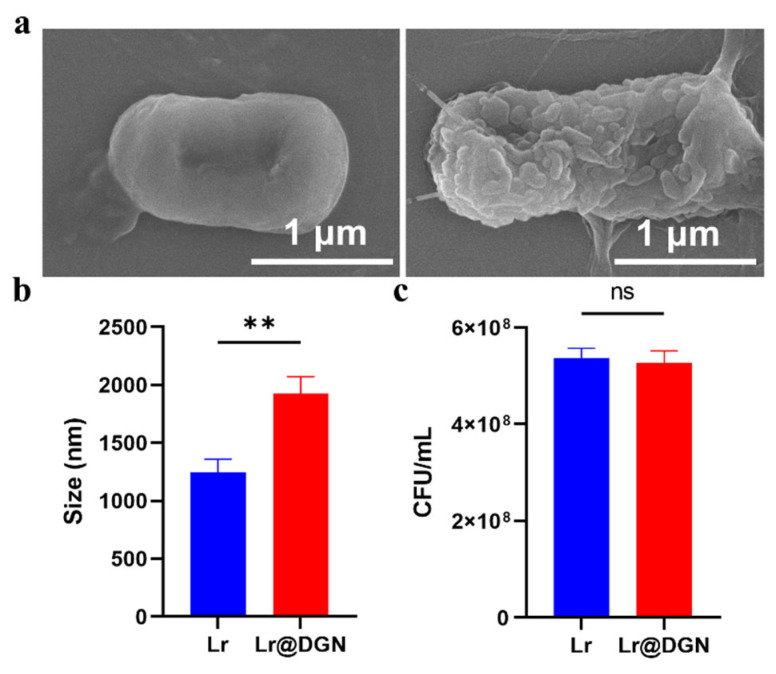
Characterization of Lr@DGN. (**a**) SEM micrographs of Lr and Lr@DGN. Scale bar, 1 μm. (**b**) Average hydrodynamic size of Lr and Lr@DGN (n = 3). (**c**) CFU counts of Lr and Lr@DGN at 24 h post-cultivation. Statistical analysis was performed using a two-sided Student’s *t*-test, with significance defined as ** *p* < 0.01. “ns” means no significance.

The success of covalent coating was visually confirmed by SEM imaging, which revealed a distinctly rougher surface on Lr@DGN compared to naive Lr (Figure 1a). Consistent with the SEM observation, DLS analysis revealed a significant increase in hydrodynamic diameter from 1247 nm (Lr) to 1926 nm (Lr@DGN), indicating successful surface conjugation (Figure 1b). Moreover, to investigate the effect of the covalent coating of DGN on the viability of Lr, quantitative analysis was made by plate counting assays, which showed no significant difference in colony-forming units (CFU) between Lr and Lr@DGN (Figure 1c). Under physiologically relevant conditions, the stability of the DGN coating was examined by incubating Lr@DGN in PBS at 37 °C for 24 h. Throughout the entire incubation period, the hydrodynamic diameter showed no significant fluctuation, and the values recorded at 24 h was close to the initial measurement (Figure S3).

### 3.2. Tolerance of Lr@DGN Under Gastrointestinal Conditions

**Figure 2:**
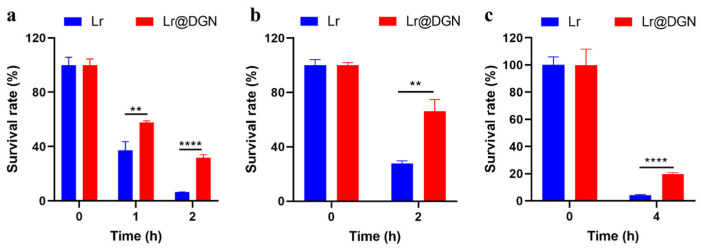
Assessing the resistance of Lr@DGN to gastrointestinal stresses. (**a**–**c**) Bacterial survival of Lr and Lr@DGN under (**a**) SGF (pH 2.5), (**b**) bile salts (4%), or (**c**) the simulated full gastrointestinal transit (2 h SGF followed by 2 h SIF). Data are summarized as mean ± SD, calculated from 3 independent biological replicates. Statistical analysis was performed using one-way ANOVA, with significance defined as ** *p* < 0.01 and **** *p* < 0.0001.

With Lr@DGN successfully prepared, we investigated whether the covalently coated DGN could enhance the resistance of Lr to gastrointestinal stresses, a key factor for its survival and colonization in the intestine. Therefore, Lr and Lr@DGN were exposed to simulated gastric fluid (SGF) and their viabilities were assessed by plate counting at 0, 1 and 2 h) following the protocol detailed in Section 2.5. Figure 2a reveals that after 2 h of incubation, the naive Lr exhibited an extremely low survival rate (6.63 ± 0.10%). In contrast, Lr@DGN, featuring a covalently bonded DGN coating, maintained a survival rate of 31.82 ± 2.25%. Compared to naive Lr, Lr@DGN demonstrated a markedly superior survival rate, representing a 4.8-fold increase. Building on these findings, we next assessed the resistance of both Lr and Lr@DGN to bile salts, another key stressor encountered in the gut [33]. As shown in Figure 2b, a similar trend was evident upon exposure to bile salts, where Lr@DGN maintained a significantly higher survival rate of 66.29 ± 8.48% after 2 h of incubation compared to only 27.36 ± 1.97% for Lr. To further assess its robustness, Lr@DGN was subjected to a sequential digestion model simulating oral administration. Following 2 h of exposure to SGF, the samples were transferred into simulated intestinal fluids (SIF; see Materials and methods for composition) for an additional 2 h incubation. As expected, Lr@DGN still exhibited a higher viability, reaching as high as 19.59 ± 1.13%, while the naive Lr was 4.17 ± 0.46% (Figure 2c).

### 3.3. In Vivo Biocompatibility Assessment of Lr@DGN After Oral Administration

**Figure 3:**
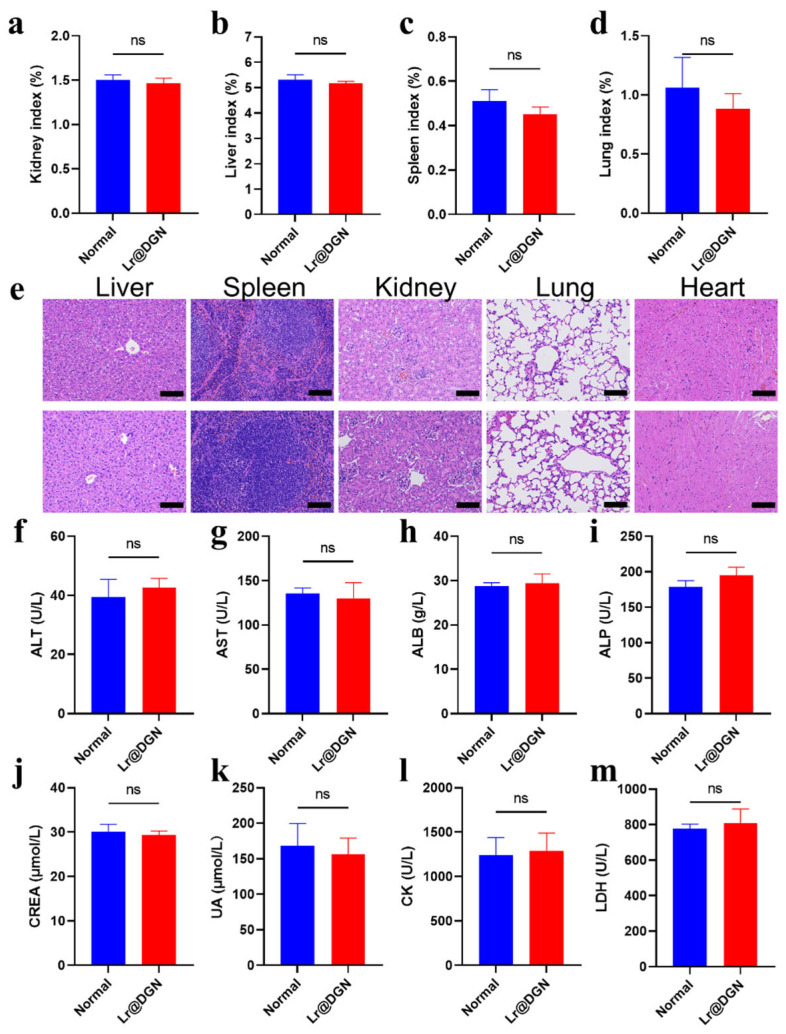
In vivo biocompatibility evaluation of Lr@DGN. (**a**–**d**) Organ indices of mice with or without oral administration of Lr@DGN. (**e**) H&E staining of major organs collected from mice following Lr@DGN administration (or PBS control). (**f**–**m**) Serum biochemistry analysis of control mice and those treated with Lr@DGN. Statistical significance was determined by two-sided Student’s *t*-test. “ns” means no significance.

Based on the encouraging findings, the in vivo performance of Lr@DGN was first evaluated, beginning with an assessment of its systemic biocompatibility. As anticipated, oral administration of Lr@DGN showed no detectable toxicity in mice. Throughout the study, key indicators of systemic health remained comparable between treated and control animals. Comprehensive monitoring included stable organ indices (kidneys, liver, spleen, lungs, and heart) (Figure 3a–d and Figure S4), as well as histopathological examination of major organs (liver, spleen, kidney, lung, and heart) by H&E staining using the methods described in Section 2.7, which revealed no signs of tissue damage or inflammation, with tissue architecture indistinguishable from that of untreated controls (Figure 3e). Furthermore, serum biochemistry parameters covering markers of liver function (alanine aminotransferase, ALT; aspartate aminotransferase, AST; albumin, ALB; alkaline phosphatase, ALP) (Figure 3f–i), kidney function (creatinine, CREA; uric acid, UA) (Figure 3j,k), and cardiac/muscle integrity (creatine kinase, CK; lactate dehydrogenase, LDH) (Figure 3l,m) showed no significant changes relative to controls.

### 3.4. Therapeutic Efficacy of Lr@DGN Toward Cy-Induced Immunosuppression in Mice

**Figure 4:**
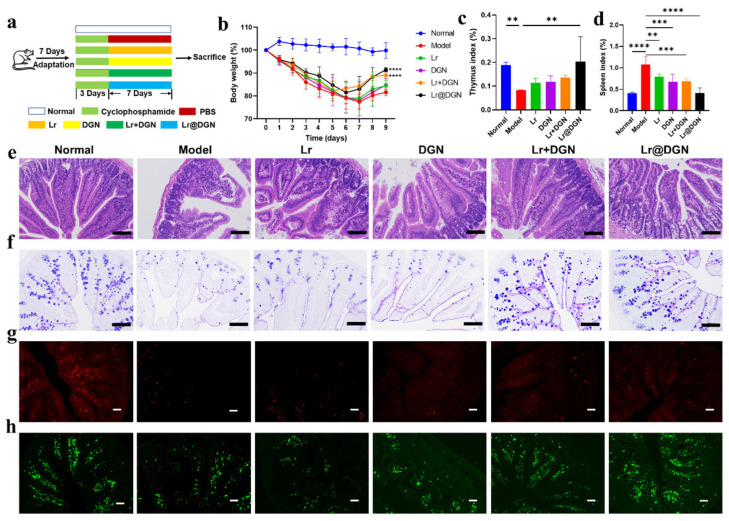
Amelioration of Cy-induced immunosuppression by Lr@DGN in mice. (**a**) Graphical illustration the therapeutic process of Lr@DGN against Cy-induced immunosuppression in mice. An immunosuppression model was established in BALB/c mice by intraperitoneal injection of Cy (80 mg/kg) for three consecutive days. Following this, the mice were orally administered Lr, DGN, Lr + DGN, or Lr@DGN daily for 7 days to evaluate the therapeutic potential of Lr@DGN. (**b**) Body weight changes over time in mice receiving different treatments. (**c**,**d**) Thymus and spleen indices of mice following various treatments. (**e**) H&E and (**f**) AB-PAS staining of small intestinal sections collected on day 10. Scale bar, 100 μm. (**g**,**h**) Immunofluorescence micrographs of CD4^+^ (**g**) and CD8^+^ T cells (**h**) in the small intestine. The red and green channels represented CD4^+^ and CD8^+^ T cells, respectively. Scale bar, 50 μm. Statistical analysis was performed using one-way ANOVA, with significance defined as ** *p* < 0.01, *** *p* < 0.001, and **** *p* < 0.0001.

Following the comprehensive characterization of Lr@DGN, we evaluated its therapeutic potential in vivo using a well-established model of Cy-induced immunosuppression. The experimental timeline is outlined in Figure 4a. Briefly, immunosuppression was induced in Balb/c mice via intraperitoneal injection of Cy (80 mg/kg body weight) for three consecutive days (days 0–2). From day 3 to day 9, the mice received daily oral gavage of PBS (model group), free DGN, free Lr, a physical mixture of Lr and DGN (Lr + DGN group), or the biohybrid Lr@DGN. A control group of healthy mice received normal water and PBS gavage (normal group).

Consistent with successful model establishment, mice in the model group exhibited a significant and sustained reduction in body weight compared to the normal group (Figure 4b). Although all treatment interventions promoted body weight recovery to some extent, only the Lr + DGN and Lr@DGN groups showed statistically significant restorative effects. Among them, the Lr@DGN group demonstrated the most pronounced recovery, showing an extremely significant difference compared to the model group (*p* < 0.0001). We next assessed the impact on primary immune organs. As expected, Cy administration caused severe thymic atrophy, reflected in a drastically reduced thymus index in the model group (*p* < 0.01, Figure 4c). Treatment with Lr@DGN significantly counteracted this atrophy, increasing the thymus index (*p* < 0.01). Furthermore, Cy induced pathological splenomegaly, likely due to compensatory hyperplasia [34]. This effect was significantly mitigated in the Lr, DGN, Lr + DGN, and Lr@DGN groups, with Lr@DGN treatment restored spleen index to levels comparable to the normal group (Figure 4d). Given the gut’s central role in immunity, we performed a detailed histopathological analysis of the small intestine tissue. H&E staining results showed that in the model group, the small intestinal mucosal structure was significantly damaged after Cy treatment, characterized by shortened villi, disordered epithelial arrangement, and a reduction in immune cells in the lamina propria (Figure 4e). Compared with the model group, treatment with Lr, DGN, or Lr + DGN all led to varying degrees of improvement in intestinal morphology. Notably, the Lr@DGN group exhibited the most pronounced restorative effect: the small intestinal mucosal structure was largely normalized, with villus height and morphology approaching normal levels, intact and continuous epithelium, a marked increase in goblet cell numbers, and significantly reduced inflammatory cell infiltration in the lamina propria (Figure 4e). The integrity of the mucosal barrier was further evaluated by assessing goblet cells via AB-PAS staining (Figure 4f). Cy administration caused a severe depletion of goblet cells in the model group. All treatments increased goblet cell numbers, with Lr@DGN proving most effective, restoring goblet cell abundance to a level indistinguishable from the normal group. Finally, to evaluate local adaptive immune reconstitution, we imaged CD4^+^ and CD8^+^ T cell populations in the small intestine via immunofluorescence as described in Section 2.12 (Figure 4g,h and Figures S5 and S6). Cy treatment significantly depleted both CD4^+^ and CD8^+^ T cells compared to the normal group. This immunosuppression was effectively reversed by Lr@DGN treatment, which significantly increased the numbers of both T cell subsets.

### 3.5. Effects of Lr@DGN on Serum Immunoglobulin Levels

Following the confirmation of Lr@DGN’s therapeutic efficacy, we proceeded to investigate its effects on serum immunoglobulin levels. Humoral immunity, a critical component of the adaptive immune system, is primarily mediated by serum immunoglobulins which play indispensable roles in pathogen neutralization and clearance [35]. As key biomarkers, IgG is the most abundant antibody isotype responsible for systemic defense against infections [36], while IgA is pivotal for mucosal immunity, providing the first line of defense at barrier surfaces [37].

**Figure 5:**
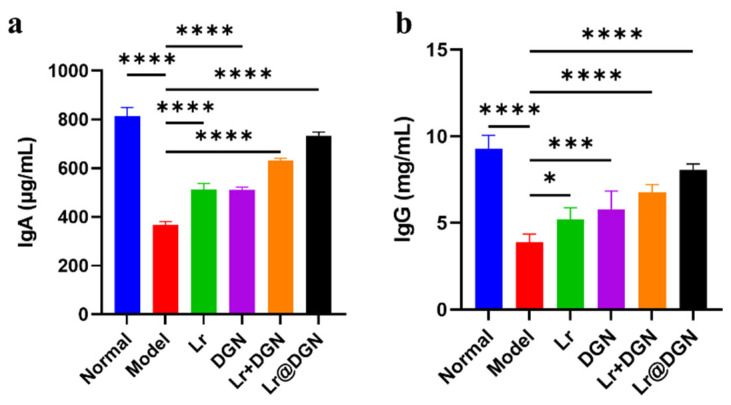
Therapeutic mechanism of Lr@DGN. Serum (**a**) IgG and (**b**) IgA levels of mice from different groups (n = 5). Statistical analysis was performed using one-way ANOVA, with significance defined as * *p* < 0.05, *** *p* < 0.001, and **** *p* < 0.0001.

We first evaluated the impact of Lr@DGN on systemic humoral immunity by measuring serum IgA and IgG levels (Figure 5a,b). As expected, Cy administration significantly suppressed both IgG and IgA production in the model group compared to the normal group. All treatment groups showed varying degrees of recovery in immunoglobulin levels. Notably, Lr@DGN treatment most effectively restored both IgG and IgA concentrations to levels comparable to the normal group, outperforming the physical mixture (Lr + DGN).

## 4. Discussion

To address the current limitations of probiotic therapies, such as low survival rates in complex physiological environments, limited colonization efficiency, and restricted immunomodulatory functions [38,39,40], this study employed a bio-orthogonal strategy to rationally design and construct a novel probiotic hybrid system, Lr@DGN. This system integrates the immunomodulatory probiotic Lr with a chemically modified GN nanoshell (DGN) that provides both physical protection and immunostimulatory activity [41]. It was specifically designed to counteract the multifaceted immunosuppressive injuries induced by Cy. The core design philosophy is twofold: first, the covalently conjugated DGN layer acts as “molecular armor”, shielding the probiotic from gastrointestinal stressors to ensure efficient intestinal delivery [41,42]; second, the interplay between viable Lr and the immunologically active DGN at the intestinal site enables multifaceted immune restoration [43,44]. Each component plays a distinct yet complementary role. Upon oral administration, the DGN layer effectively protects Lr from erosion by gastric acid and bile salts. After reaching the intestine, the viable Lr and DGN act in concert at the gut–immune interface. Through multiple pathways, they collectively work to restore both systemic and mucosal immune homeostasis.

In vivo evaluation in a Cy-induced immunosuppression mouse model confirmed the superior therapeutic efficacy of Lr@DGN. Oral administration of Lr@DGN was significantly more effective than free Lr, free DGN, or their physical mixture in alleviating body weight loss and restoring thymus and spleen indices to normal levels. Histological analysis further demonstrated that Lr@DGN effectively repaired Cy-induced damage to the intestinal mucosal architecture. This included restoring villus height and morphology, increasing goblet cell numbers, reducing inflammatory infiltration, significantly promoting the recovery of local intestinal CD4^+^ and CD8^+^ T cell populations. Furthermore, Lr@DGN effectively reversed Cy-induced suppression of serum IgG and IgA production. These results directly reflect the integrated functional advantages of this hybrid system.

The Lr@DGN system offers several advantages over traditional probiotic delivery methods and recent biohybrid platforms. Conventional encapsulation techniques like hydrogels, microcapsules, and enteric coatings focus on protecting probiotics from gastrointestinal stressors [45]. While they enhance survival, these methods often rely on inert materials that do not contribute to therapeutic effects. In contrast, our “functional protection” strategy uses DGN as a dual-purpose shell, providing both protection and immune support through its prebiotic and immunostimulatory properties. This approach aligns with the growing trend of using delivery materials as active therapeutic agents. Compared to other biohybrid systems, such as those with polydopamine coatings [46] or metal–phenolic networks [47], our system offers unique benefits. The copper-free bio-orthogonal click chemistry allows covalent conjugation under mild conditions, preserving bacterial viability without cytotoxic catalysts. Additionally, the DGN shell provides both physical protection and immune modulation, offering integrated benefits not typically seen with single-function coatings. In a Cy-induced immunosuppression model, Lr@DGN demonstrated superior recovery in body weight, organ indices, intestinal mucosal integrity, T cell populations, and immunoglobulin levels compared to free components or physical mixtures. These features position Lr@DGN as a promising next-generation biohybrid system combining material design and therapeutic function.

While the results are promising, several limitations must be considered when evaluating the scalability and clinical translation of Lr@DGN. In terms of scalability, the current synthesis is based on laboratory-scale bacterial culture and bio-orthogonal conjugation. Scaling up to industrial production would require optimizing fermentation conditions for high-density bacterial cultures, improving conjugation efficiency to lower material costs, and establishing reliable quality control systems to ensure consistency between batches. Furthermore, the stability of the covalent conjugation during long-term storage, such as in lyophilization or when formulated with excipients, has yet to be systematically tested before commercialization. Regarding clinical translation, the efficacy data were obtained using a Cy-induced immunosuppression mouse model. Although this model is well-established, it does not fully replicate the complexities of human immunosuppressive conditions. Additional evaluation in larger animal models, assessment of the immunogenicity of the DGN shell or conjugation chemistry, and toxicology studies conducted under good laboratory practice guidelines are necessary before advancing to clinical use. Furthermore, the oral formulation might need further development to improve stability or achieve targeted release. Overcoming these challenges will be essential for moving Lr@DGN toward clinical applications.

In summary, Lr@DGN represents a next-generation integrated probiotic-based therapeutic strategy. Within a single, rationally designed platform, it combines the gut-modulating and immune-activating properties of probiotics with the enhancement offered by an immunostimulatory adjuvant. The enhanced therapeutic efficacy of Lr@DGN can be explained mechanistically by the synergistic effects of its components. Specifically, the DGN shell likely activates the innate immune system through its interaction with the Dectin-1 receptor on antigen-presenting cells [48]. On the other hand, the viable Lr core contributes to the maintenance of intestinal barrier integrity and the regulation of local immune homeostasis, primarily by producing short-chain fatty acids and interacting directly with the gut-associated lymphoid tissue [49]. The covalent conjugation of the components ensures that both elements are delivered in a coordinated manner to the gut–immune interface, facilitating their cooperative effects. This is further supported by the superior therapeutic performance of Lr@DGN compared to the physical mixture. To gain a more comprehensive understanding of the molecular mechanisms driving its immunomodulatory activity, further investigations into its underlying mechanisms are necessary. These will involve approaches such as cytokine profiling, immune cell subset analysis, and pathway inhibition experiments, which are crucial for a complete elucidation of the process. Nonetheless, this work provides a robust conceptual foundation and translational insight for the design of integrated probiotic biohybrid systems with enhanced therapeutic efficacy.

## Data Availability

The original contributions presented in this study are included in the article/Supplementary Materials. Further inquiries can be directed to the corresponding author.

## Supplementary Materials

The following supporting information can be downloaded at https://www.mdpi.com/article/10.3390/foods15091540/s1, Figure S1: The zeta potential of DGN and carboxylated GN (n = 3). Figure S2: Flow cytometry analysis of DBCO-AF647 labeling. Lr-N₃ (azido-labeled) and unmodified Lr were incubated with DBCO-AF647, followed by flow cytometry analysis (n = 3). Figure S3: Average hydrodynamic size of Lr@DGN at 0 h and 24 h (n = 3). Figure S4: Heart index of mice with or without oral administration of Lr@DGN (n = 3). Figure S5: Immunofluorescence micrographs of CD4^+^ T cells in the small intestine. The red channels represented CD4^+^ T cells. Figure S6: Immunofluorescence micrographs of CD8^+^ T cells in the small intestine. The green channels represent CD8^+^ T cells.
