# Supplementary material for: Prebiotic-Empowered Probiotics with Gastrointestinal Stress Resistance for Enhanced Oral Therapy of Immunosuppression
Source: Foods. 2026 Apr 29;15(9):1540. doi: 10.3390/foods15091540 (PMC13164192; doi:10.3390/foods15091540)
Supplement: Supplementary file 1 [file foods-15-01540-s001.zip › foods-4216534-supplementary.pdf]

Supplemental Information

Prebiotic-empowered probiotics with gastrointestinal stress resistance for enhanced oral therapy of immunosuppression

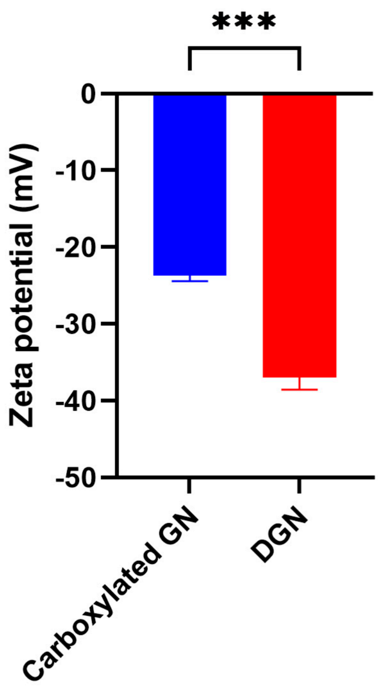

Figure S1. The zeta potential of DGN and carboxylated GN (n=3).

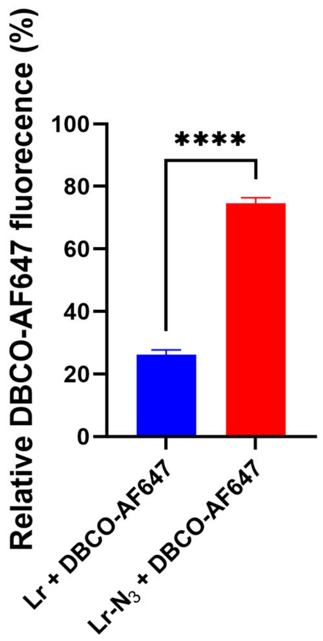

Figure S2. Flow cytometry analysis of DBCO-AF647 labeling. Lr-N_3 (azido-labeled) and unmodified Lr were incubated with DBCO-AF647, followed by flow cytometry analysis (n=3).

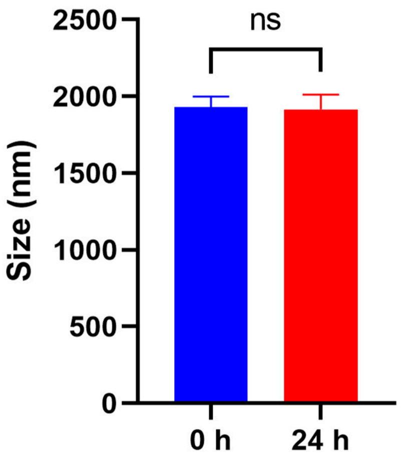

Figure S3. Average hydrodynamic size of Lr@DGN at 0 h and 24 h (n=3).

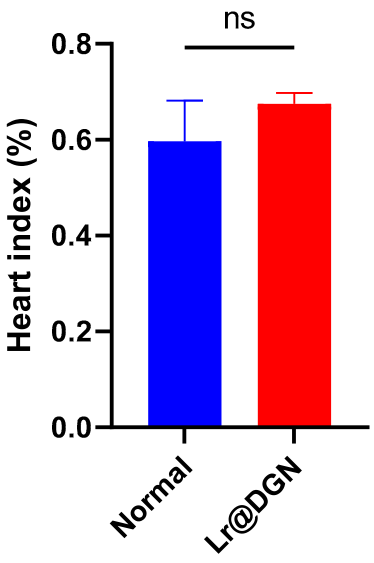

Figure S4. Heart index of mice with or without oral administration of Lr@DGN (n=3).

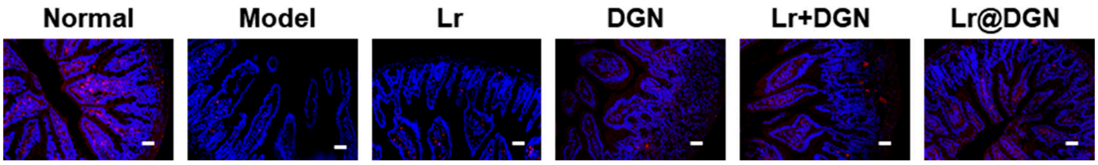

Figure S5. Immunofluorescence micrographs of $CD4^+$ T cells in the small intestine. The red channels represent $CD4^+$ T cells.

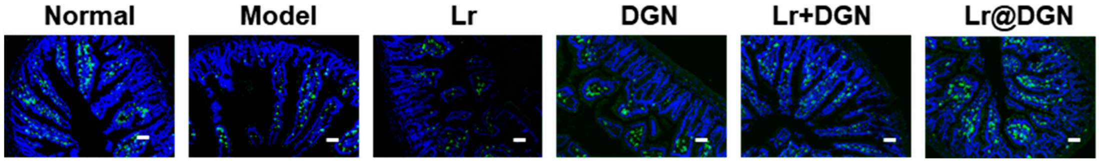

Figure S6. Immunofluorescence micrographs of $CD8^+$ T cells in the small intestine. The green channels represent $CD8^+$ T cells.
